# Optimized enzymatic colorimetric assay for determination of hydrogen peroxide (H_2_O_2_) scavenging activity of plant extracts

**DOI:** 10.1016/j.mex.2015.05.001

**Published:** 2015-05-18

**Authors:** Chamira Dilanka Fernando, Preethi Soysa

**Affiliations:** Department of Biochemistry & Molecular Biology, Faculty of Medicine, University of Colombo, Kynsey Road, Colombo 08, Sri Lanka

**Keywords:** Enzymatic colorimetric assay for H_2_O_2_ scavenging activity, Colorimetric assay, Hydrogen peroxide, Scavenging activity, Plant extracts

## Abstract

The classical method to determine hydrogen peroxide (H_2_O_2_) scavenging activity of plant extracts is evaluated by measuring the disappearance of H_2_O_2_ at a wavelength of 230 nm. Since this method suffers from the interference of phenolics having strong absorption in the UV region, a simple and rapid colorimetric assay was developed where plant extracts are introduced to H_2_O_2_, phenol and 4-aminoantipyrine reaction system in the presence of horseradish peroxidase (HRP). This reaction yields a quinoneimine chromogen which can be measured at 504 nm. Decrease in the colour intensity reflects the H_2_O_2_ scavenged by the plant material.

• Optimum conditions determined for this assay were 30 min reaction time, 37 °C, pH 7, enzyme concentration of 1 U/ml and H_2_O_2_ concentration of 0.7 mM. The limit of detection (LOD) and limit of quantitation (LOQ) were 136 μM and 411 μM, respectively.
• Half maximal effective concentration required to scavenge 50% of H_2_O_2_ in the system (EC_50_ value) calculated for several plant extracts and standard antioxidants resulted in coefficient of variance (CV%) of the EC_50_ values less than 3.0% and correlation coefficient values (*R*^2^) > 0.95 for all dose response curves obtained.
• This method is convenient and very precise which is suitable for the rapid quantification of H_2_O_2_ scavenging ability of standard antioxidants and natural antioxidants present in plant extracts.

## Graphical abstract

**Figure:**
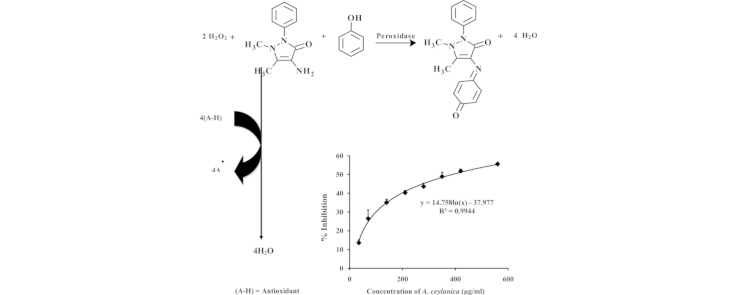

## Method details

### Background information

**Fig. 1:**
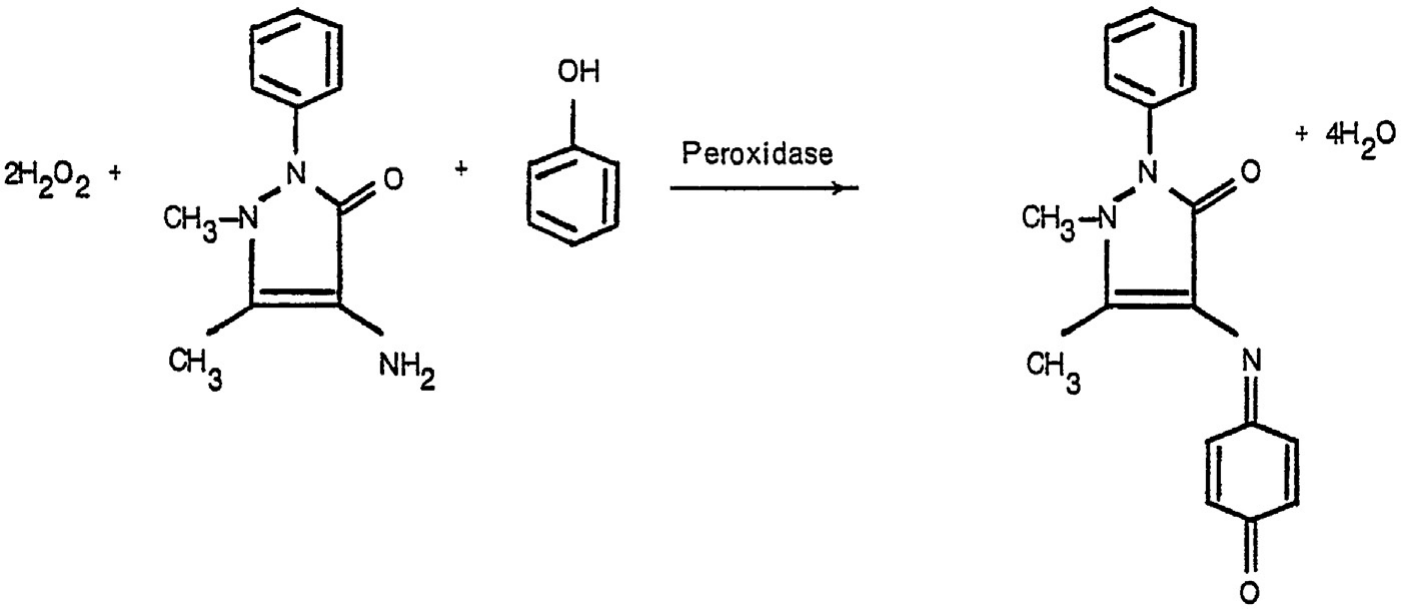
The chemical reaction catalyzed by HRP [8].

Hydrogen peroxide (H_2_O_2_) scavenging activity of natural antioxidants present in plant extracts has been determined widely [1], [2], [3], [4], [5] by measuring decrement of H_2_O_2_ in an incubation system containing H_2_O_2_ and the scavenger using the classical UV-method at 230 nm [6]. The main disadvantage of this method is the possible interference from secondary metabolites present in plants which absorb in UV region [7]. Therefore, a simple and rapid colorimetric assay was developed to determine H_2_O_2_ scavenging activity of plant extracts and standard antioxidants based on the reaction system where H_2_O_2_ rapidly reacts with phenol and 4-aminoantipyrine in the presence of horseradish peroxidase (HRP) to produce a pink coloured quinoneimine dye (Fig. 1) [8]. H_2_O_2_ scavengers will eventually result in decreased production of this particular chromophore. This method was applied to standard antioxidants ascorbic acid, gallic acid and tannic acid in addition to selected plant extracts to determine their hydrogen peroxide scavenging abilities.

### Chemicals and equipment

The chemicals gallic acid, 4-aminoantipyrine and horse radish peroxidase (HRP) were purchased from Sigma Chemicals Co. (P.O. Box 14508, St. Louis, MO 63178, USA). l-Ascorbic acid and hydrogen peroxide were purchased from BDH Chemicals (BDH Chemicals Ltd Poole, England). Tannic acid was purchased from Riedel De Haen Ag, Wunstorfer Strasse 40, SEELZE1, D3016, Germany. Phenol was purchased from Fluka (Fluka chemie GmbH, CH-9471, Buchs, Switzerland). Plant extracts were freeze dried using LFT 600EC freeze dryer. SHIMADZU UV 1601 UV Visible spectrophotometer (Shimadzu Corporation, Kyoto, Japan) was used to measure the absorbance.

### Plant materials

*Atalantia ceylanica* (Yaki-naran), *Eriocaulon quinquangulare* (Heen kokmota) and *Semecarpus parvifolia* (Heen badulla) were collected from Anuradhapura, Kalutara and Colombo districts, respectively. *Mollugo cerviana* (Pathpadagam) total plant was purchased from a traditional medicinal drug store. *Camellia sinensis* Crush-Tear-Curl (CTC) black tea powder was purchased from the local market. Plants were identified and confirmed by Department of Botany, Bandaranaike Memorial Ayurvedic Research Institute, Nawinna, Sri Lanka.

### Preparation of the plant extracts

All the plant materials *A*. *ceylanica* (leaves), *E*. *quinquangulare* (whole plant), *M*. *cerviana* (whole plant), *S*. *parvifolia* (leaves) were prepared separately as decoctions according to the proportions followed by Ayurvedic practitioners. The plant materials described above were washed separately with tap water followed by distilled water and de-ionized water, dried to achieve a constant weight. Each plant material was cut into small pieces and ground to a fine powder using a clean kitchen blender. Powdered samples (30 g) except *S*. *parvifolia* were boiled with 800 ml of deionized water until the total volume reduced to 100 ml (1/8th of the original volume) using a beaker. Powdered leaves of *S*. *parvifolia* (30 g) was refluxed with 800 ml of deionized water to prepare the aqueous extract of the plant material. The decoctions were sonicated and filtered through a cotton wool plug and then using filter paper (Whatman No. 1). The filtrates were centrifuged at 2000 rpm for 10 min. The supernatants were freeze dried. The freeze dried samples were weighed, and stored at −20 °C in sterile tubes until further use. A weight of 2.0 g of *C*. *sinensis* (black tea powder) was added into 200 ml of boiling water and allowed to stand in a closed beaker to prepare black tea infusion. The solution was allowed to cool to room temperature, filtered through filter paper (Whatman No. 1) and the filtrate was used for the experiments.

### Determination of incubation period, enzyme concentration and H_2_O_2_ concentration

A mixture containing phenol (12 mM, 350 μl), 4-aminoantipyrene (0.5 mM, 100 μl), H_2_O_2_ (0.7 mM, 160 μl) and phosphate buffer at pH 7 (84 mM, 350 μl) was prepared in separate tubes for each incubation period (5–60 min) to optimize the incubation time needed for the completion of the reaction. HRP (0.1 U/ml, 40 μl) was added to each tube and incubated at 37 °C. At the end of the each incubation period the absorbance was measured at 504 nm against reagent blank consisting of phosphate buffer instead of phenol.

Varying concentrations of HRP (0.01–1 U/ml) were incubated with substrates at pH 7 for 30 min. at 37 °C as described above to optimize the enzyme concentration needed for the assay.

Similarly different concentrations of H_2_O_2_ (0.175–0.70 mM), phenol, 4-aminoantipyrene, phosphate buffer (pH 7) were incubated with HRP (1 U/ml) as described above and the absorbance was read to study the best H_2_O_2_ concentration to be used for the assay.

**Fig. 2:**
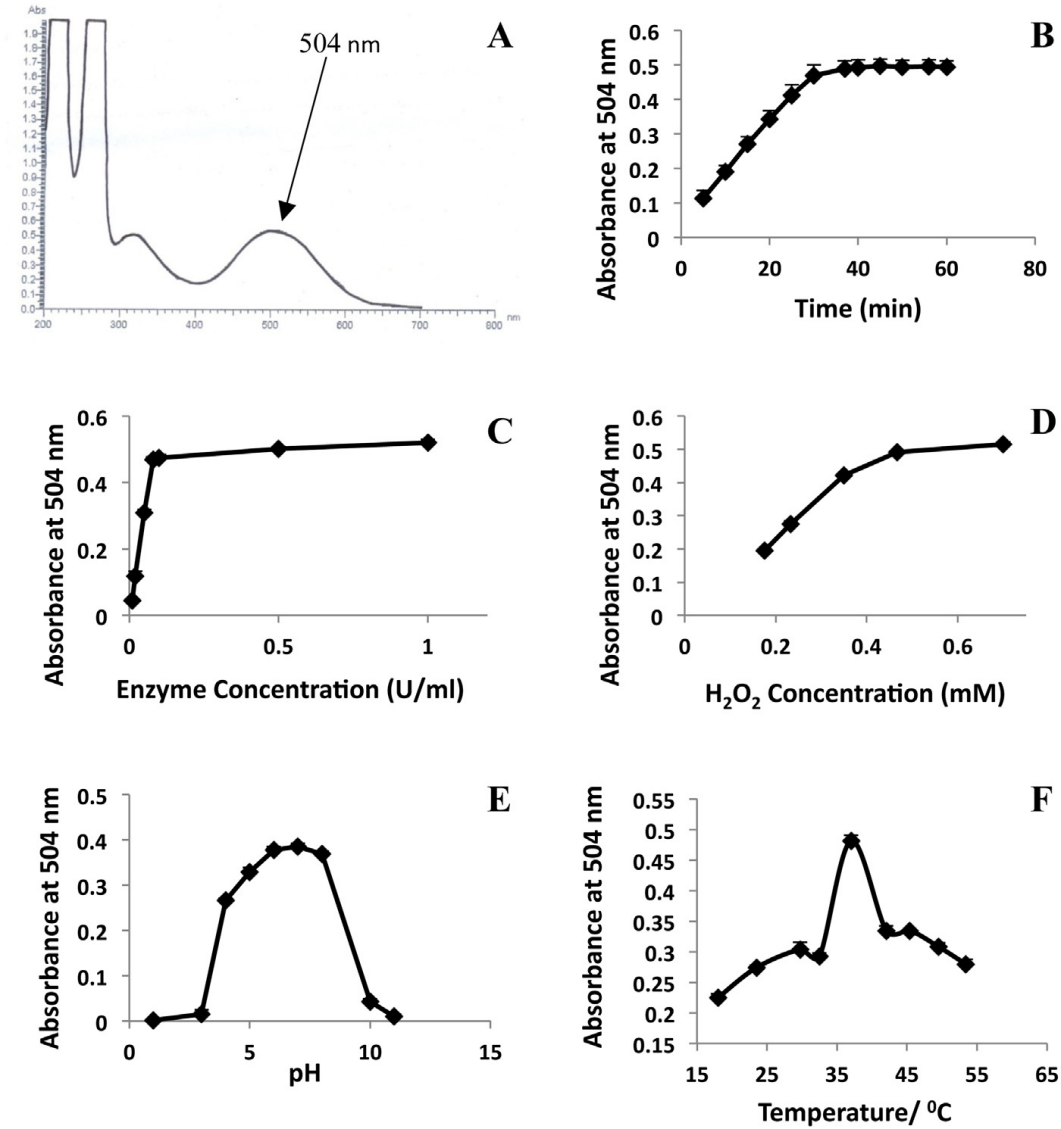
The UV–vis spectrum for the chromogen formed at wave length range from 200 to 800 nm (A), variation of absorbance with time (B), variation of absorbance with enzyme concentration (C), dependance of absorbance on H_2_O_2_ concentration (D), pH stability of the chromogen formed (E) and variation of absorbance with temperature (F). The results are presented as mean + SD of three independent experiments.

In the present colorimetric method to determine H_2_O_2_ scavenging activity, maximum wavelength of absorbance (*λ*_max_) of the quinoneimine dye at pH 7 was observed at 504 nm (Fig. 2A). At 37 °C, pH 7, H_2_O_2_ concentration of 0.7 mM and horse radish peroxidase enzyme concentration of 0.1 U/ml, maximum intensity of the quinoneimine dye was resulted at 30 min and the colour was stable until 60 min (Fig. 2B). When solutions having enzyme concentrations of 0.01–1 U/ml (maintained at 37 °C, pH 7 with H_2_O_2_ concentration of 0.7 mM) were incubated for 30 min, the colour increased drastically from enzyme concentration of 0.01–0.08 U/ml but very slight increment of the colour was observed from 0.08 to 1 U/ml (Fig. 2C). Although enzyme concentration of 0.1 U/ml turned out to be suitable for the experiment, further studies were carried out keeping enzyme concentration 10 folds higher (1 U/ml) to compensate for possible inhibition of enzyme that can be caused by phytochemicals present in plant extracts. When varying the H_2_O_2_ concentration from 0.175 to 0.70 mM of the solutions having enzyme concentration of 1 U/ml (maintained at pH 7, 37 °C for an incubation period of 30 min), it was observed that the enzyme is being saturated at H_2_O_2_ concentration of 0.70 mM (Fig. 2D) and therefore this concentration was used for the assay.

### Determination of optimum pH and temperature

Mixtures containing phenol (12 mM, 350 μl), 4-aminoantipyrene (0.5 mM, 100 μl), H_2_O_2_ (0.7 mM, 160 μl) and phosphate buffer at pH 7 (84 mM, 350 μl) were prepared in separate tubes and pH was varied from 1 to 11. HRP (1 U/ml, 40 μl) was added to each tube and was incubated at 37 °C for 30 min. The absorbances of the resulting solutions were measured at 504 nm against the reagent blank consisting of phosphate buffer at pH 7 instead of phenol.

Substrates were incubated with HRP (1 U/ml) at different temperatures (18–54 °C) at pH 7 for 30 min and absorbance was measured at 504 nm as described above.

When pH was varied from 1 to 11 of the solutions having H_2_O_2_ and peroxidase concentrations of 0.7 mM and 1 U/ml, respectively, maintained at 37 °C and incubated for 30 min, maximum intensity of colour of the resultant dye was yielded at pH 7 (Fig. 2E). When temperature was varied from 18 to 54 °C of the solutions maintained at similar conditions as above and at pH 7, maximum intensity of the colour of the dye was observed at 37 °C (Fig. 2F).

### Determination of limit of detection (LOD) and limit of quantitation (LOQ)

The LOD and LOQ values were determined according to the ICH guidelines provided [9]. Microsoft Excel was used to perform regression analysis of the calibration curve constructed using diluted samples of H_2_O_2_. The standard deviation of the *y*-intercepts (*δ*) of the regression line and the slope of the calibration curve (S) were estimated. LOD and LOQ were calculated as 3.3 × *δ*/S and 10 × *δ*/S, respectively [9]. The calculated values were 136 μM and 411 μM for LOD and LOQ, respectively.

### Determination of H_2_O_2_ scavenging activity

According to the above experiments the optimum conditions selected for the reaction catalyzed by horse radish peroxidase is stated in Table 1. These conditions were maintained when plant extracts were introduced into these systems to assess their scavenging ability of H_2_O_2_ molecules. Phenol (12 mM) and 4-aminoantipyrene (0.5 mM) were chosen and used for all the above tests as these concentrations led to maximum intensity of the resultant chromophore. The percentage inhibition (% I) of H_2_O_2_ caused by plant extracts and standard antioxidants was calculated as follows. Reaction mixture comprising of test sample (plant extract/standard antioxidant; 350 μl), phenol solution (12 mM, 350 μl), 4-aminoantipyrene (0.5 mM, 100 μl), H_2_O_2_ (0.7 mM, 160 μl) and HRP (1 U/ml) prepared in phosphate buffer (84 mM, pH 7) was incubated at 37 °C for 30 min. The absorbances of the resulting solutions were measured at 504 nm against reagent blank consisting of phosphate buffer instead of plant extract/standard antioxidant and phenol. The control was made out of same reagents except plant extract replaced by phosphate buffer. Interference for the assay from the plant extracts was minimized as follows. For each concentration of plant extract, samples for background subtraction were made using the plant extract with other reagents replacing phenol by phosphate buffer. Each resulting absorbance value was subtracted from the relevant original absorbance reading. Five types of plant extracts known for their antioxidant properties were tested for their H_2_O_2_ scavenging activities. l-Ascorbic acid, gallic acid and tannic acid were used as reference standard antioxidants. The percentage inhibition of hydrogen peroxide was calculated by the equation as described as for many antioxidant assays [3], [4], [5]:

$$\%Inhibition=\frac{\text{Abs. of control}-\text{Abs. of sample}}{\text{Abs. of control}}\times 100\%$$

The effective concentration required to scavenge 50% of H_2_O_2_ in the system (EC_50_ value) was calculated from either linear or logarithmic dose response curves plotted between % Inhibition of hydrogen peroxide versus concentration of test samples/standards. EC_50_ values were presented as mean ± standard deviation (Mean ± SD) of six independent experiments. Student’s *t*-test was used to compare mean EC_50_ values of standard antioxidants/plant extracts and *p* value <0.05 was considered as significant. Coefficient of variance (CV %) was computed for the EC_50_ values obtained for plant extracts/standard antioxidants. All regression and statistical analyses were performed using Microsoft Excel software.

**Fig. 3:**
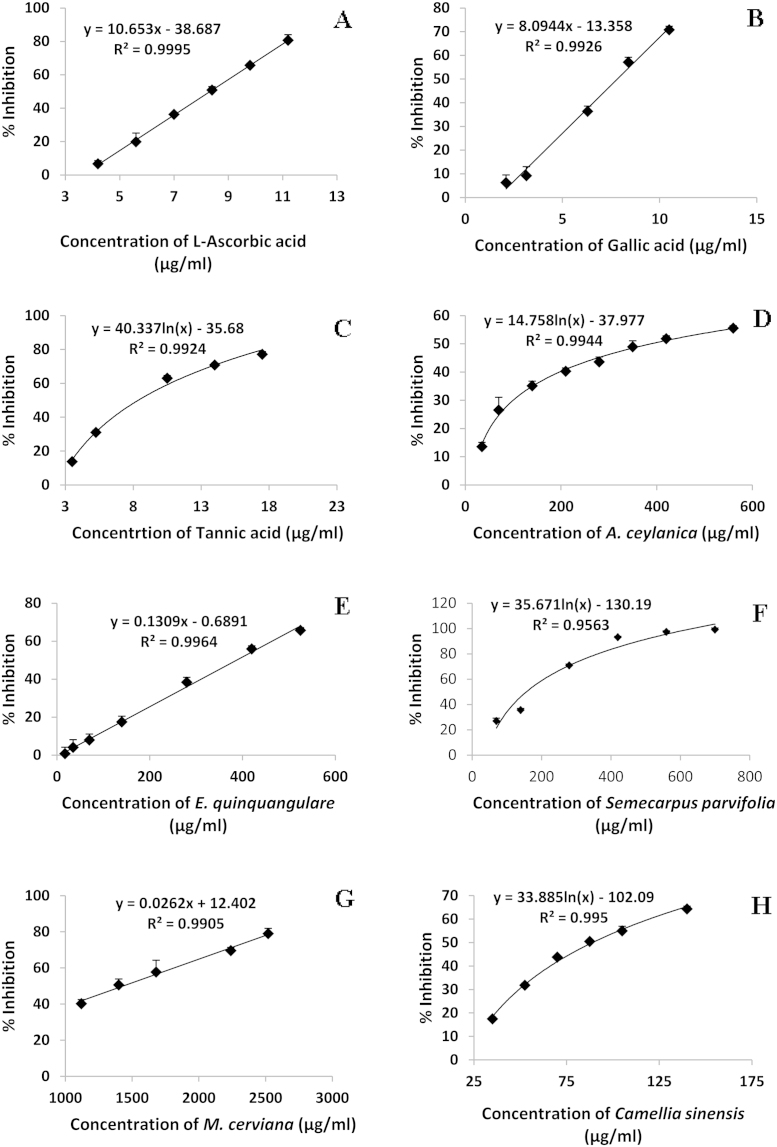
The dose response curves for percentage inhibition (% I) of hydrogen peroxide by l-ascorbic acid (A)*,* Gallic acid (B), tannic acid (C) standard antioxidants, *A*. *ceylanica* (D), *E*. *quinquangulare* (E) decoctions, *S*. *parvifolia* aqueous extract (F) *M*. *cerviana* decoction (G) and *C*. *sinensis* infusion (H). The results are presented as mean + SD of six independent experiments. Correlation coefficient values (*R*^2^) exceeded 0.95.

In this study, various plant extracts and standard antioxidants were investigated for their hydrogen peroxide scavenging ability utilizing the developed method by comparing the EC_50_ (half maximal effective concentration) values obtained from the corresponding dose response curves via linear or logarithmic regression analyses (Fig. 3A–H). The amount of chromogen formed in the reaction between H_2_O_2_, phenol and 4-aminoantipyrine (catalyzed by HRP) decreased in a dose dependant manner of the plant extracts/standard antioxidants due to their scavenging ability of H_2_O_2_ molecules.

*C*. *sinensis* black tea infusion had the highest ability to scavenge H_2_O_2_ molecules followed by *S*. *parvifolia*. There was no significant difference in H_2_O_2_ scavenging ability between *A*. *ceylanica* and *E*. *quinquangulare* (*p* > 0.05) but both of these extracts had lesser H_2_O_2_ scavenging ability than *S*. *parvifolia*. *M. cerviana* had the least ability to scavenge hydrogen peroxide molecules. However, the scavenging ability of hydrogen peroxide was superior in the standard antioxidants (i.e., l-ascorbic acid, gallic acid and tannic acid) than all the plant extracts studied. H_2_O_2_ scavenging ability of l-ascorbic acid was significantly lower (*p* < 0.001) than gallic acid and tannic acid. There was no significant difference in H_2_O_2_ scavenging ability observed between gallic acid and tannic acid (*p* > 0.05).

In addition, we have conducted most widely used antioxidant assay i.e.; DPPH test [10] for some of the above mentioned plant extracts and antioxidants. With respect to this assay, the antioxidant potential for these substances varied according to l-ascorbic acid > *C*. *sinensis* *>* *S*. *parvifolia* *>* *A*. *ceylanica* *>* *M. cerviana* [10], [11], [12], [13]. Similar pattern of variation in the antioxidant potential of the same substances was observed in the method developed for the determination of H_2_O_2_ scavenging activity.

For the current method for analysis of H_2_O_2_ scavenging activity, coefficient of variance (CV%) for the EC_50_ values obtained were less than 3.0% for all the plant extracts and standard antioxidants studied. The correlation coefficient values (*R*^2^) of the dose response curves were greater than 0.95 (Table 2).

When considering other methods used for the determination of H_2_O_2_ scavenging activity, the classical UV-method is widely used where the decrement of H_2_O_2_ in an incubation system containing H_2_O_2_ and the scavenger is measured at 230 nm. According to this method described by Ruch et al. [6], we experienced fluctuations in absorbance. This encountered with less reproducible and reliable results. The interference from secondary metabolites present in plants which absorb in UV region affects the results [7]. The present method involves background correction for endogenous interfering substances which improves the reproducibility. Czochra and Widénska [14] have developed fluorescence spectroscopic method using homovanillic acid (4-hydroxy-3-methoxyphenylacetic acid) and peroxidase for the determination of H_2_O_2_ scavenging activity of plant extracts and standard antioxidants. Although it is a highly selective and sensitive method, fluorescence generated can be lost by quenching, resonance energy transfer and inner filter effect in the presence of various endogenous phytochemicals apart from the fact that fluorescence spectrometry being a costly method [15]. UV–vis spectrophotometers are widely used in most of the laboratories in low-income countries, and the current method can be used without additional burden. Zhang [16], has estimated hydrogen peroxide scavenging activity of plant extracts by replacement titration method. This method described based on iodide oxidation by hydrogen peroxide is a time consuming macro method where a titration is involved and may not be feasible with higher number of analytes [17]. Many different systems too have been described and are commercially available for quantification of H_2_O_2_ in experimental systems, for instance, chemiluminescent, fluorogenic substrates like Amplex Red [18], chromogenic substrates like tetramethylbenzidine (TMB) [19] and 2,2′-azino-bis(3-ethylbenzothiazoline-6-sulphonicacid) (ABTS) [20]. The reaction of peroxidase catalyzed conversion of H_2_O_2_, phenol and 4-aminoantipyrine to a chromogenic substance is utilized in many commercially available assay kits designed for bio-analytical tests which include determination of blood glucose [21] and cholesterol [22]. However this reaction is not involved so far for the determination of H_2_O_2_ scavenging activity of various antioxidants. Therefore use of the same reagents for determination of H_2_O_2_ scavenging activity gives a dual purpose for the reagents which in turn reduces costs for the purchase of special chemicals like Amplex red, TMB and ABTS.

In conclusion, the colorimetric method developed and optimized in the current study for the quantification of H_2_O_2_ scavenging activity of standard antioxidants as well as natural antioxidants present in plant extracts is less expensive, precise, rapid and yields reproducible results. Therefore this method is ideal for routine laboratory analyses.

## Additional information

During aerobic metabolism as well as in the process of drug biotransformation, reactive oxygen species (ROS) are produced as by-products. These include radicals such as superoxide anion (O_2_ **•^−^**), hydroxyl radical (HO•), alkoxyl radical (RO•), peroxyl radical (ROO•) and non radicals such as hydrogen peroxide (H_2_O_2_) and singlet oxygen (^1^O_2_) [23]. ROS can cause lipid oxidation, protein oxidation, DNA strand breaks, and modulation of gene expression. Experimental evidences show that these ROS are involved in liver diseases and also lead to atherosclerosis, cancer, stroke, asthma, arthritis and other age related diseases [24]. In order to combat ROS, living organisms have developed defense mechanisms consisting of variety of antioxidant enzymes such as superoxide dismutase, catalase, glutathione peroxidase, glutathione reductase [25] as well as non-enzymatic antioxidants such as ascorbic acid, α-tocopherol, β-carotene, flavonoids and many phenolic compounds [26]. Antioxidants are compounds that when present in low concentration in relation to the oxidant prevent or delay the oxidation of a particular oxidizable-substrate [27]. Since natural antioxidants are capable of scavenging various ROS, many methods have been developed for the estimation of these properties.

Hydrogen peroxide can be formed in vivo by various oxidizing enzymes such as superoxide dismutase. It can permeate through biological membranes slowly oxidizing number of compounds. Hydrogen peroxide is used in the respiratory burst of activated phagocytes [28]. Although hydrogen peroxide itself is not very reactive [29], it can generate the highly reactive hydroxyl radical (HO•) through the Fenton reaction [30] and is found to be main reason for toxicity associated with hydrogen peroxide. Hydrogen peroxide can deactivate enzymes involved in cellular energy production such as glyceraldehyde-3-phosphate dehydrogenase found in glycolytic pathway [31] as well as aconitase and α-ketoglutarate dehydrogenase found in Krebs cycle [32] by oxidation of essential thiol (—SH) groups. Therefore, scavenging of hydrogen peroxide is considered as an important feature of antioxidants [33]. Accepting electrons in the presence of electron donors, hydrogen peroxide is decomposed into water [34]. Hydrogen peroxide scavenging activity especially of phenolic compounds is assigned to their electron-donating ability [35].

## Figures and Tables

**Table 1:** Selected optimum conditions for the reaction catalyzed by horse radish peroxidase enzyme.

Parameter	Optimum value
Incubation time	30 min
Enzyme concentration	1 U/ml
H_2_O_2_ concentration	0.7 mM
Temperature	37 °C
pH	7

**Table 2:** The EC_50_ values, coefficient of variance (CV %), regression equations and correlation coefficients (*R*^2^ values) obtained for dose response curves of various plant extracts and reference standard antioxidants using the developed H_2_O_2_ scavenging activity test.

Sample (*n* = 6)	EC_50_ value (μg/ml) mean ± SD	CV %	Regression equation	*R*^2^ value
*A*. *ceylanica* (Yakinaran)	388.11 ± 4.11	1.1	*y* = 14.75 ln(*x*) − 37.97	0.994
*E*. *quinquangulare* (Heen kokmota)	381.98 ± 1.83	0.5	*y* = 0.130*x* − 0.689	0.996
*S*. *parvifolia* (Heen badulla)	156.25 ± 2.85	1.8	*y* = 35.67 ln(*x*) − 130.1	0.956
*M*. *cerviana* (Pathpadagam)	1480.3 ± 43.1	2.9	*y* = 0.026*x* + 12.40	0.990
*C*. *sinensis* (black tea)	91.96 ± 2.51	2.7	*y* = 33.88 ln(*x*) − 102.0	0.995
l-Ascorbic acid	10.0 ± 0.14	1.4	*y* = 10.65*x* − 38.68	0.999
Gallic acid	7.82 ± 0.19	2.4	*y* = 8.094*x* − 13.35	0.992
Tannic acid	8.17 ± 0.10	1.2	*y* = 40.33 ln(*x*) − 35.68	0.992

EC_50_ = half maximal effective concentration, SD = standard deviation, *y* = percentage inhibition (% I) of H_2_O_2_, *x* = concentration (μg/ml), CV % = coefficient of variance %, *R*^2^ = correlation coefficient.
